# Templating the morphology of soft microgel assemblies using a nanolithographic 3D-printed membrane

**DOI:** 10.1038/s41598-020-80324-y

**Published:** 2021-01-12

**Authors:** John Linkhorst, Jonas Lölsberg, Sebastian Thill, Johannes Lohaus, Arne Lüken, Gerhard Naegele, Matthias Wessling

**Affiliations:** 1 AVT.CVT - Chair of Chemical Process Engineering, RWTH Aachen University, Forckenbeckstraße. 51, 52074 Aachen, Germany; 2 DWI - Leibniz Institute for Interactive Materials, Forckenbeckstr. 50, 52074 Aachen, Germany; 3 Biological Information Processing (IBI-4), Forschungszentrum Jülich, Jülich, 52425 Germany

**Keywords:** Gels and hydrogels, Chemical engineering, Mechanical engineering

## Abstract

Filter cake formation is the predominant phenomenon limiting the filtration performance of membrane separation processes. However, the filter cake’s behavior at the particle scale, which determines its overall cake behavior, has only recently come into the focus of scientists, leaving open questions about its formation and filtration behavior. The present study contributes to the fundamental understanding of soft filter cakes by analyzing the influence of the porous membrane’s morphology on crystal formation and the compaction behavior of soft filter cakes under filtration conditions. Microfluidic chips with nanolithographic imprinted filter templates were used to trigger the formation of crystalline colloidal filter cakes formed by soft microgels. The soft filter cakes were observed via confocal laser scanning microscopy (CLSM) under dead-end filtration conditions. Colloidal crystal formation in the cake, as well as their compaction behavior, were analyzed by optical visualization and pressure data. For the first time, we show that exposing the soft cake to a crystalline filter template promotes the formation of colloidal crystallites and that soft cakes experience gradient compression during filtration.

## Introduction

Particle deposition and pore clogging of porous media are encountered in a large variety of industrial filtration processes such as water purification or product separation. These phenomena often denoted as fouling, represent a serious limitation to the filtration performance. In pressure-driven membrane filtration, colloidal particles deposit on the membrane surface, forming a filter cake^1,2^. This cake layer often controls the transport and rejection of certain species in the feed, effectively taking over the role of the actual membrane^3^.

Hard particles commonly form amorphous, isotropic filter cakes, whose morphology is independent of the applied transmembrane pressure. These hard-particle filter cakes can be well described with classical colloidal filtration theories^1,4^. However, the filtration of soft particles is more complex. Soft particles can undergo deformation, interpenetration, and compression^5^, such that the resulting filter cakes deform partly reversibly and irreversibly during filtration^6^. Additionally, the particles in a soft cake can rearrange their position due to deformation and often create crystalline cake regions, such that the cake becomes strongly non-isotropic^7–9^. Therefore, conventional cake filtration models often lead to erroneous prediction, e.g., of the filtration rate when applied to soft filter cakes^4,10^.

Since many technically relevant filtration processes contain soft biological matter, it is essential to understand the fundamental behavior of soft filter cakes. A significant challenge is to perform non-invasive measurements on the pore-scale level and within the filter cake, to understand the processes happening at the single particles. Microfluidics has proven to be a powerful tool to tackle this challenge.

Microfluidic systems provide well-defined custom geometries, typically realized using transparent elastomer^11^. This allows the in-situ observation of artificial membrane pores using optical analysis methods. Several studies have been conducted to gain a better understanding of pore clogging, backwashing effects and cake filtration in microfluidic filtration setups^2,12–19^.

In previous work^14^, we showed how hard, fluorescently labeled colloidal particles permeate through a filter cake built up from soft microgels. Akin to the static experiments in previous works, the microgel particles form crystalline and amorphous regions in the filter cake^7–9^. We found that the crystalline regions in the filter cake facilitate the permeation of the colloids^14^. Consequently, controlling the morphology of the filter cake to enhance filtration performance is highly desirable.

The present work investigates (1) whether one can influence the soft cake morphology by a surface templating of the artificial, rigid membrane and (2) how the soft particles are compressed inside the soft cake. Therefore, we developed a method to fabricate a microfluidic crystal template as the rigid model membrane to manipulate the cake morphology. The traditional soft lithography approach to manufacture microfluidic chips is extended by 3D printing a rigid template structure into a microchannel using nanolithography^20^. The template is designed as a hexagonally close packed crystal, consisting of four layers of connected rigid spheres (${6\,}\upmu \text{m}$). Soft microgels of $6\,\upmu \text{m}$ (diameter) dispersed in water are filtered towards this template structure, forming a filter cake. We use CLSM to analyze the three-dimensional cake morphology and deformability of the filter cake. This enables us to answer whether the structure of a template can manipulate the morphology of the filter cake and how the soft filter cake is compressed towards the membrane.

## Results and discussion

### Additive manufacturing of colloidal crystals

In order to create a self-supporting crystal of individual spheres that do not disintegrate after printing, measures were taken to increase structural integrity. In the crystal, the particles only have point contact, meaning that the spheres only touch each other at a single point. Printing this would not result in a crystal but individual spheres. A convenient way to strengthen the crystal is by inserting smaller, interconnecting spheres in the contact point. The size of the interconnecting spheres is designed in a way that does not significantly influence the pore size of the crystal.

**Figure 1:**
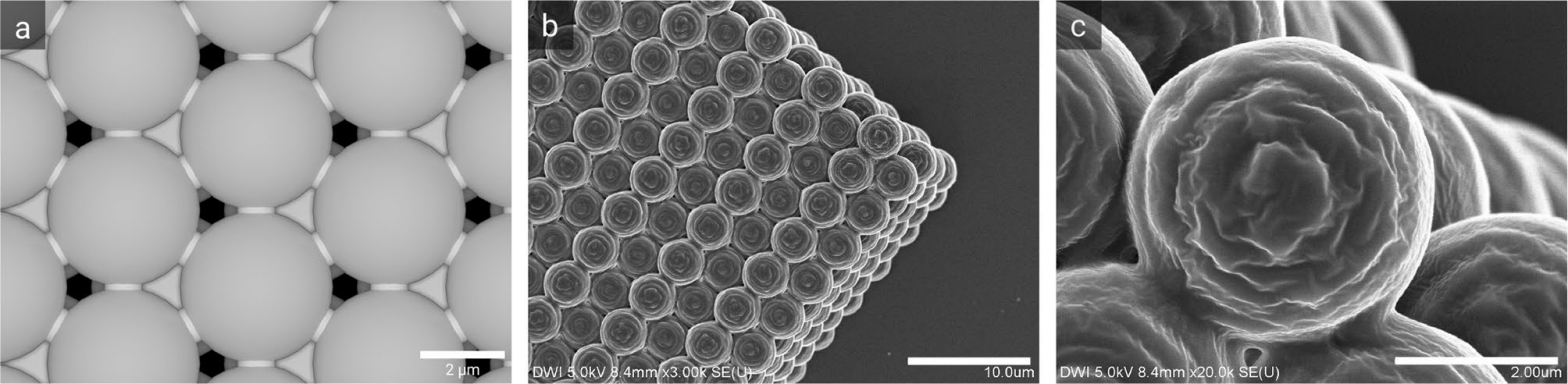
(**a**) Rendered computer model of an HCP crystal, stabilized with interconnecting small spheres positioned at the contact position between the larger crystal spheres (Blender). SEM images of a 3D nanolithographic printed colloidal HCP crystal at (**b**) 3k and (**c**) 20k magnification. The spheres are well interconnected, and the pores in between are open, enabling solvent permeation through the crystal.

Figure 1a shows an image of a sphere-connected crystalline layer, rendered in the open-source software Blender. The pores are open, and the interconnecting spheres are merged into the crystal, forming reinforced bridges. Using this design, colloidal crystals have been manufactured with 3D nanolithography. Figure 1 shows SEM images of a 3D printed colloidal crystal following the Hexagonal Close Packing (HCP) lattice with $30 \times 15 \times 7$ spheres with a diameter of $3.6\,\upmu \text{m}$. The SEM images show that the spheres are well interconnected and that the interstitial pores are open. Figure 1b,c show that the production method of printing the spheres by stacking concentric circles leaves visible artifacts on the surface. To reduce this effect and match the size of the sphere to that of the later employed compressed microgels, the sphere size was selected as $5\,\upmu \text{m}$ for the template design.

### Microfluidic templating chip

**Figure 2:**
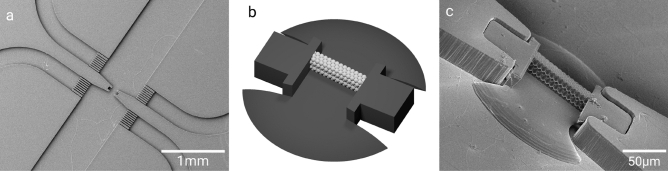
(**a**) SEM image of the center of the microfluidic chip without the template. (**b**) CAD model of the template used for the in-chip fabrication. (**c**) SEM image of the crystal template, printed into the microfluidic chip. To produce the SEM images in (**a**) and (**c**), the channel was left open during the print, and not bonded to a cover glass.

Having accomplished the fabrication of rigid, free-standing HCP crystals, we integrated them into a microfluidic chip with connected tubing and fluid control. In this way, the templating of soft particles in front of the printed crystal becomes possible. Figure 2a shows the microfluidic chip before 3D printing, designed with a pocket to hold the consecutively imprinted template. Six fluid channels ensure that the chip can be efficiently flushed with solvent after printing and simplify preparing the experiments.

**Figure 3:**
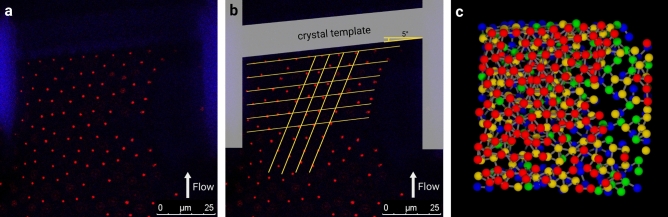
(**a**) CLSM image of the microgel filter cake in front of the rigid template (in blue). The red dots are the fluorescently labeled cores of the microgels. The shells are not visible, allowing for the differentiation of neighboring particles. (**b**) CLSM image (**a**), overlayed with straight yellow lines that indicate the filter cake’s orientation and crystal symmetry lines. For clarity, the slightly tilted crystal template is highlighted in the image (in grey). (**c**) Computer-generated 3D image of the extracted center positions of the microgels. The microgel spheres are differently colored for different horizontal layers, starting from blue for the bottom layer, green, yellow, and red (top layer). The gray bars, connecting neighboring microgels, are introduced as a guide to the eye. Visualization was performed via the python interface of Blender 2.8 (www.Blender.org).

Since crystal formation also occurs without templates^14^, the printed template was slightly tilted by $5^{\circ }$ away from the vertical to the channel symmetry line (compare Fig. 3b) to be able to differentiate between natural crystal formation and particle templating in the experiments (Fig. 2b). The support structure for the crystal is shown in dark gray. On the left and right side of the crystal, the interlocking blocks were designed to fit into the pocket and thereby prevent the crystal from being flushed out during experiments. The wing-shaped bottom plates serve as sealing and improve adhesion to the channel. To minimize its effect on the flow profile, the bottom plate has a flat dome shape.

Figure 2c shows an electron microscopy image of a successful print. For illustration purposes, the imprint was performed using an open chip. It shows that the $5^{\circ }$ angle is retained in the chip and held by the interlocking blocks, while the crystal itself is well integrated into the microfluidic chip. While the final chip is stabilized with a glass slide, the open chip is soft and shows a slight difference in height between the PDMS and the imprint that is supposedly caused by misalignment due to the elasticity of the material. This was not observed in the closed chip used in the experiments.

### Filter cake morphology in a microfluidic templating chip

Building on the above results, the microfluidic PDMS channel containing the imprinted crystal template was used during all experiments. A soft filter cake was formed by flushing an aqueous suspension of spherical core-shell microgels in front of the template. After building up the filter cake in the chip, we studied its morphology.

Figure 3a,b show the same z-slice of confocal data for a partially crystalline filter cake. The red dots are the fluorescent cores of the microgels, while the non-fluorescent shell remains invisible. The autofluorescence of the printing resin is visible in pale blue. On the right side of the figure, the filter cake is more dilute since a dust particle (not visible) blocks a part of the channel. In Fig. 3b, a bungle of straight yellow lines is drawn in parallel to the tilted template. Another set of four straight lines is drawn under a $60^{\circ }$ angle relative to the first bundle, a crystal symmetry axis, as a guide to the eye. As the lines indicate, the filter cake is partially crystalline, since the particles roughly align with the lines. Between the crystalline patches, grain boundaries and intermediate amorphous regions are visible. The crystalline patches are tilted by $5^{\circ }$, such as the template. Interestingly enough, the crystalline order, tilted by $5^{\circ }$, is retained even across grain boundaries and amorphous regions, which can be seen from the image.

In previous work^14^, we were also able to detect colloidal crystals in the filter cake. However, in the vicinity of the channel walls, the cake was found to be amorphous, and the crystallinity was developed only upstream. This earlier finding is fundamentally different from the present work, employing templates, where crystalline patches are observed directly at the template surface. Even though the template-induced soft crystalline cake is far from being uniform, it nicely demonstrates that using a crystalline template enables to form crystalline domains and to manipulate their orientation. This shows that the membrane surface structure can template the cake morphology. From our earlier work, we anticipate a uniform mono-crystal formation for a larger template surface. Being able to remove the limitation of the printing volume in the future will allow creating larger template areas.

Figure 3c shows a computer-generated 3D image of the soft microgel filter cake based on the confocal image stack. Four horizontal layers of microgel spheres are consecutively colored by their respective height in the channel, with blue being the bottommost layer, green and yellow for the intermediate layers, and red for the top layer.

### Visualization of deformation gradient by compression and relaxation

**Figure 4:**
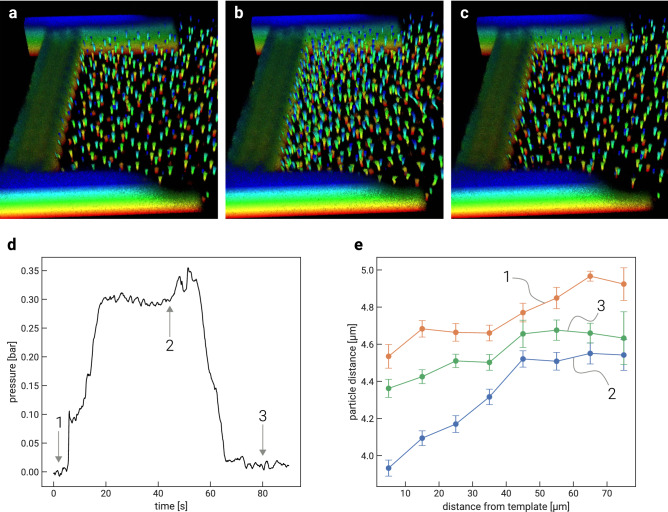
3D CLSM time series of the consecutive compression and relaxation of the microgel filter cake. (The color code for the horizontal layers is the inverse the one of Fig. 3): (**a**) uncompressed reference state; (**b**) compressed state at 0.3 bar (TMP); (**c**) relaxed state at 0 bar to prove reversibility; (**d**) measured pressure during the experiment; (**e**) average next-neighbor particle distance of the states 1, 2, and 3 as a function of the distance from the template surface (error bars shows normalized standard deviation).

Figure 4 shows frames of a 3D time series of the compression and relaxation experiment, with pressure data and distance analysis. Figure 4a marks the reference point of the experiment with a relaxed filter cake. This filter cake has a low crystallinity as the particles were jammed into the view field. Figure 4b shows the compressed state at 0.3 bar applied pressure. To ensure steady-state conditions, the image was taken after maintaining the pressure for 40 s. The increased pressure leads to an inhomogeneous particle density across the filter cake. The particle concentration decreases from a high value at the rigid template towards lower values upstream. To prove truly reversible compression, the pressure was released, with the filter cake relaxing close to its reference state (Fig. 4c).

From the three confocal data sets in Fig. 4a–c respectively, particle coordinates and next-neighbor distances have been determined and plotted as a function of the distance from the template (Fig. 4e). Figure 4d shows the corresponding pressure during the compression experiment. Graphs 1 and 3 describe a nearly homogeneous particle-particle distance with only a slight compression slope. The development between graphs 1 and 3 indicates a rearrangement within the filter cake towards higher crystallinity, allowing for higher particle concentrations. Curve 2 shows a steady increase with inter-particle distance up to $\sim 45\,\upmu \text{m}$ distance from the rigid template and flattens further upstream. This behavior conforms with the expected pressure distribution in the channel, were the strongest pressure gradients are in the most narrow sections. While in previous work^9^, we were able to identify the shape of deformation of individual particles in the filter cake, we can now visualize the gradient formation of filter cakes under filtration conditions on the scale of the particles. To the best of our knowledge, while such behavior could be expected, it was not visualized on the particle scale before the present work.

## Conclusion and outlook

We fabricated intricate hard colloidal 3D crystals via in-chip nanolithography in microfluidic chips and conducted experiments on templating soft microgel crystals at these crystal filter templates. The template was tilted by $5^{\circ }$ to differentiate between natural crystal formation and templating induced one. The filter cake built up by the filtration of soft microgels followed the angle of the template by forming crystallites that were also tilted by $5^{\circ }$. This proves that the orientation of the filter cake crystals can be programmed by integrating filter templates. Furthermore, we showed that soft filter cakes experience a gradient compression, stemming from the drag force exerted on the particles that add up towards the template, effectively resulting in stronger particle compaction closer to the template. While filter cake compaction is a well-know phenomenon in membrane science, the deformation gradient formation of filter cakes has never been visualized on this small scale before. Having 3D position data available now will serve to validate future simulations of filter cake compression of soft particles.

With the ability to create templates with well-defined morphology even down to the particle scale, filter cakes can be structurally modified. In future developments, this knowledge can be profitably used for filtration process optimization. Templating offers the opportunity to exploit the orientation-dependent properties of the soft crystalline cake for filtration purposes. Hexagonal Close Packing (HCP) crystals could be templated in a way such that the pinholes are parallel to the flow direction to add an additional size-exclusion layer to a membrane. One benefit of such a crystalline size-exclusion layer is that internal clogging or irreversibly attached fouling material can be removed via backwashing together with the crystal, leaving the membrane still intact.

## Materials and methods

### Chemicals

The microgels used in this study are 500 nm (diameter) trifluoroethyl methacrylate (TFMA) particles with an N-isopropylacrylamide-co-acrylic acid (p(NIPAM-co-AAc)) shell. The core is labeled with fluorescent dye Nile Red^21^. The microgel concentration in the dispersion was set to 1 mg/mL.

Due to the carboxyl groups in the p(NIPAM-co-AAc) chains, the particles are pH-sensitive. This is exploited to size the microgels in the dispersion to $6\,\upmu \text{m}$ (diameter). When increasing the solution’s pH-value, the deprotonation of the COOH groups to $\text{COO}^{-}$ leads to an increase of repelling among the chains and thus to an increase in the diameter of the particle. To match the diameter of the template, the pH-value of the solution was raised to pH 4. To accomplish this, a 0.1 M sodium-hydroxide (NaOH) solution was added to the solution until pH 4 was reached. The ion concentration is so low that the microgels practically act as soft electroneutral particles.

### Analytics and manufacturing

To visualize the nanolithographic printing results, scanning electron microscope (SEM) images are taken with a Hitachi S-4800 FE-SEM device. Optical analysis of the filter cake is performed with a Leica SP8 Confocal Laser Scanning Microscope (CLSM). The images were analyzed with imageJ for feature tracking, and custom python scripts were used for calculating inter-particle distances and creating the graphs.

The microfluidic chip was prepared using the standard soft lithography replica molding PDMS process. The PDMS slab was bonded to a $175\,\upmu \text{m}$ cover glass to keep in the focus range for in-chip fabrication.

The crystalline structures to be imprinted were designed and programmed using the unique python API of the graphics software Blender. The templates were produced via nanolithography. The structures were prepared and written through the cover glass directly into the microfluidic chips with a Photonic Professional GT (Nanoscribe GmbH) 3D printer using the accompanying DeScribe (file preparation) and NanoWrite (object writing) software. Prior to printing, the chips were filled with IP-L 780 photoresist (Nanoscribe GmbH). The prints were developed with acetonitrile (ACN) and subsequently flushed first with isopropanol and then water.

The template structures from Fig. 1 were then written into the pocket of the microfluidic chip, according to the method described by Loelsberg et al.^20^. The chip is flushed with IP-L 780 resin and mounted on the stage of the Photonic Professional GT printer, and the origin of the 3D-file, which is the starting position for the print, is found manually in the chip. The x and y origin can be found optically with the camera of the 3D printer and the pocket walls. The z origin is found with the help of the physical properties of the printing resin, which slightly fluoresces at the focal point when excited with laser light. If a low intensity is chosen, the resin is not crosslinked, and the difference in emitted light of the resin and the PDMS chip can be observed to find the interface and the z origin for printing. The structure is then written into the channel. After the chip has been unmounted, the print is developed by carefully flushing with acetonitrile, a strong solvent for the printing resin that swells PDMS by only 1%^22^. The low swelling ratio ensures that the pocket is still tight enough to hold the crystal during the development step.

### Experimental conduct

In the first type of experiments (templating a crystalline filter cake), core-shell microgels have been accumulated in front of the printed crystal. We flushed a dense dispersion through the crossflow channels, which allows positioning the particles right in front of the template. The filter cake build-up occurred in the second step when the crossflow channels were closed, and a dead-end filtration through the main channel was conducted by applying a constant flow through the imprinted crystal. The filter cake morphology was then observed optically via CLSM.

The second type of experiment was designed to study the gradient deformation of the filter cake. In a first step, a reference measurement with the same filter cake build-up as before was taken in a relaxed state at 0 bar. Gradient compression is studied by a pressure-step experiment. The pressure is increased to 0.3 bar and kept constant for 40 s to allow the particles to reach their steady-state position. The pressure is measured using a strain gauge that is glued onto the cover glass of the microfluidic chip. The pressurized channel expands slightly, such that the strain gauge changes its resistance. The actual pressure is calculated by calibration curves before the experiment. To verify true dynamic compaction, a third measurement is taken after relaxation to reach similar particle distances as the reference measurement.
